# Variation in arterial input function in a large multicenter computed tomography perfusion study

**DOI:** 10.1007/s00330-021-08067-6

**Published:** 2021-05-28

**Authors:** Daan Peerlings, Edwin Bennink, Jan W. Dankbaar, Birgitta K. Velthuis, Hugo W. A. M. de Jong, On behalf of the DUtch acute STroke (DUST) study investigators, C. B. Majoie, Y. B. Roos, L. E. Duijm, K. Keizer, A. van der Lugt, D. W. Dippel, K. E. Droogh - de Greeve, H. P. Bienfait, M. A. van Walderveen, M. J. Wermer, G. J. Lycklama à Nijeholt, J. Boiten, D. Duyndam, V. I. Kwa, F. J. Meijer, E. J. van Dijk, F. O. Kesselring, J. Hofmeijer, J. A. Vos, W. J. Schonewille, W. J. van Rooij, P. L. de Kort, C. C. Pleiter, S. L. Bakker, J. Bot, M. C. Visser, B. K. Velthuis, I. C. van der Schaaf, J. W. Dankbaar, W. P. Mali, T. van Seeters, A. D. Horsch, J. M. Niesten, G. J. Biessels, L. J. Kappelle, M. J. Luitse, Y. van der Graaf

**Affiliations:** 1 Department of Radiology, University Medical Center Utrecht, Utrecht, 3584CX The Netherlands; 2 Image Sciences Institute, University Medical Center Utrecht, Utrecht, 3584CX The Netherlands

**Keywords:** Stroke, Tomography, X-ray computed, Perfusion imaging, Contrast media

## Abstract

**Objectives:**

To report the variation in computed tomography perfusion (CTP) arterial input function (AIF) in a multicenter stroke study and to assess the impact this has on CTP results.

**Methods:**

CTP datasets from 14 different centers were included from the DUtch acute STroke (DUST) study. The AIF was taken as a direct measure to characterize contrast bolus injection. Statistical analysis was applied to evaluate differences in amplitude, area under the curve (AUC), bolus arrival time (BAT), and time to peak (TTP). To assess the clinical relevance of differences in AIF, CTP acquisitions were simulated with a realistic anthropomorphic digital phantom. Perfusion parameters were extracted by CTP analysis using commercial software (IntelliSpace Portal (ISP), version 10.1) as well as an in-house method based on block-circulant singular value decomposition (bSVD).

**Results:**

A total of 1422 CTP datasets were included, ranging from 6 to 322 included patients per center. The measured values of the parameters used to characterize the AIF differed significantly with approximate interquartile ranges of 200–750 HU for the amplitude, 2500–10,000 HU·s for the AUC, 0–17 s for the BAT, and 10–26 s for the TTP. Mean infarct volumes of the phantom were significantly different between centers for both methods of perfusion analysis.

**Conclusions:**

Although guidelines for the acquisition protocol are often provided for centers participating in a multicenter study, contrast medium injection protocols still vary. The resulting volumetric differences in infarct core and penumbra may impact clinical decision making in stroke diagnosis.

**Key Points:**

*• The contrast medium injection protocol may be different between stroke centers participating in a harmonized multicenter study.*

*• The contrast medium injection protocol influences the results of X-ray computed tomography perfusion imaging.*

*• The contrast medium injection protocol can impact stroke diagnosis and patient selection for treatment.*

**Supplementary Information:**

The online version contains supplementary material available at 10.1007/s00330-021-08067-6.

## Introduction

The computed tomography perfusion (CTP) protocol is central to a large number of multicenter stroke studies. These studies often focus on the impact of endovascular or intra-arterial therapies on stroke outcome and use CTP as a selection modality [1–3]. A premise in these studies is that the CTP data from the contributing centers is uniform in diagnostic quality and quantitative results (e.g., infarct core volume), and can be pooled to form a homogeneous database.

However, the CTP protocol involves a number of technical acquisition and processing steps that may violate this assumption of uniformity [4, 5]. This heterogeneity may lead to significantly different (quantitative) results, necessitating harmonization of the acquisition and processing steps.

Studies have already shown that variation in the injection protocol can influence CTP results. The effect of contrast medium factors and patient factors on the time attenuation curve has been studied with a physiologically based pharmacokinetic model [6]. Also in a patient study, some of these contrast medium and patient factors were found to affect the time attenuation curve [7]. Furthermore, it was shown that a higher iodine contrast concentration can improve the quality of patient perfusion data [8]. Although several aspects of the injection protocol have been deliberated, the clinical variation between centers participating in a harmonized multicenter study and the effect this variation can have on the perfusion analysis have not been studied.

This paper explores the variation in contrast injection protocol, as characterized by the arterial input function (AIF), for centers participating in a multicenter CTP study to test the hypothesis that substantial differences in CTP results arise.

## Methods

**Fig. 1:**
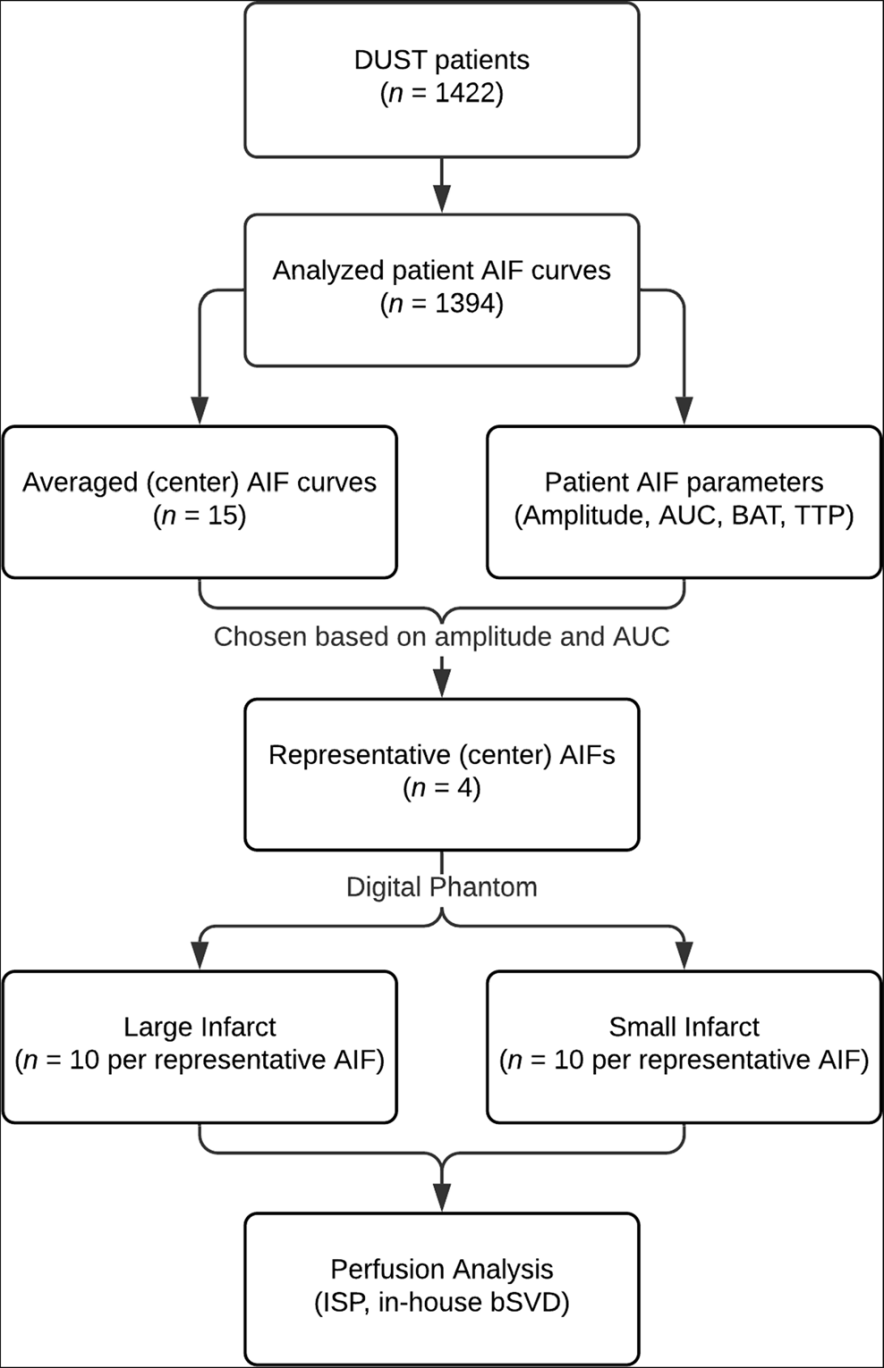
Flowchart of our study. AIF stands for arterial input function, AUC stands for area under the curve, BAT stands for bolus arrival time, and TTP stands for time to peak

The methods of our study follow the steps visualized in Fig. 1.

### Acquisition of imaging data

Imaging data was acquired from fourteen stroke centers (labelled center A-M) that participated in the DUtch acute STroke (DUST) study [9]. Patients with a clinical diagnosis of acute ischemic stroke were included if they were older than 18 years, if they had an acute neurological deficit of less than 9 h, and if the National Institutes of Health Stroke Scale was at least 2 or, if an indication for intravenous recombinant tissue plasminogen activator was present, was equal to 1.

The DUST study protocol design describes acquisition at 80 kVp and 150 mAs on 40- to 320-detector CT scanners (GE Healthcare, Philips, Siemens, Toshiba) with a 2-s interval for a duration of 50 s and reconstructed to a slice thickness of 5 mm. The advised injection protocol was a 40 mL contrast bolus injected at a rate of 6 mL/s followed by a saline flush of 40 mL injected at a rate of 6 mL/s.

### Determination of acquisition protocols

Although a general CTP acquisition protocol was formulated for the centers participating in the DUST study, the acquisition protocols still varied between centers. Since these protocols were not inventoried at the time of the DUST study and we were unable to retrieve them retrospectively, the acquisition protocols were reconstructed from the imaging data.

Whereas the scan protocols could be reproduced from the DICOM metadata of the CTP scans, information about the injection protocol was not stored in the DICOM dataset. In order to still study the injection protocol, we looked at the variation in AIF, which reflects all important aspects of the injection protocol.

### Determination of patient AIFs

All CTP data were processed centrally in a uniform manner. Prior to analysis, the scans were corrected for motion by three-dimensional registration on the skull using the registration software package Elastix [10]. For each scan, the AIF was determined from the registered image employing in-house software by averaging all attenuation curves in an automatically segmented part of the arterial tree of at least a hundred voxels. The AIF was then rescaled, such that the area under the curve (AUC) of the AIF equaled the AUC of the automatically segmented venous output function (VOF), to correct for partial volume effects. Contrary to clinical practice, the automatically determined AIF was never manually rectified.

### Processing of patient AIFs

**Fig. 2:**
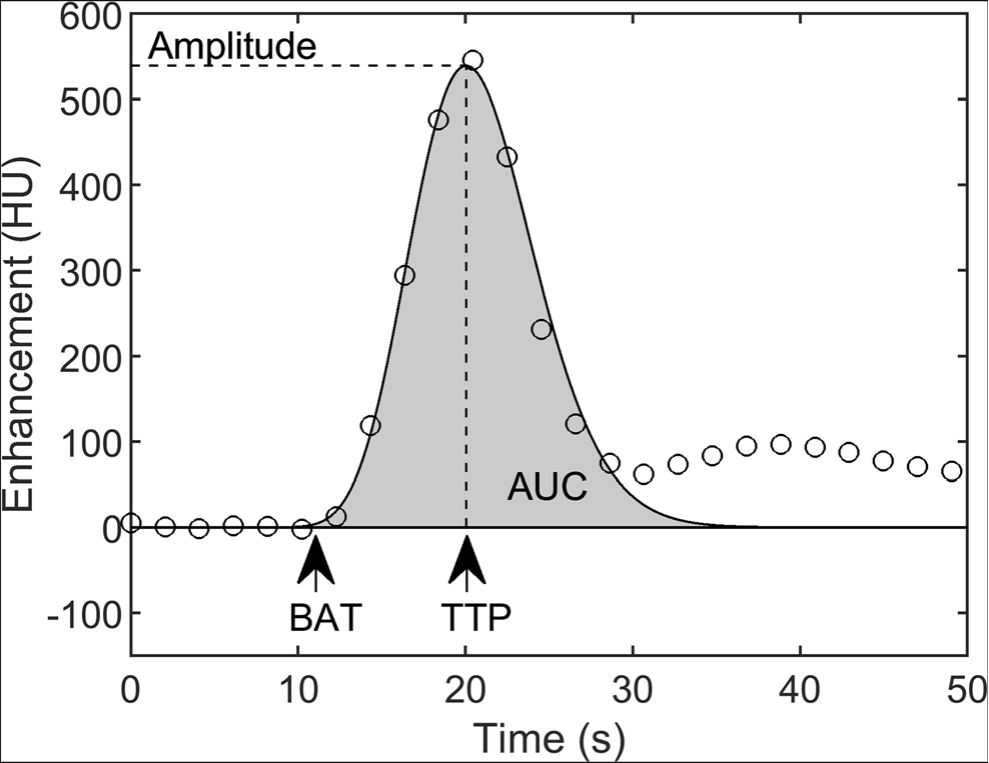
The considered parameters of the arterial input function: amplitude, area under the curve (AUC), bolus arrival time (BAT), and time to peak (TTP). The BAT is defined as the left 0.05% percentile of the maximum of the gamma distribution curve

For each AIF, the amplitude, AUC, bolus arrival time (BAT), and time to peak (TTP) were automatically determined from a gamma distribution fitted to the AIF (Fig. 2). A boxplot was made for each parameter to show the variation within and between centers.

To test for significant differences between centers, a one-way analysis of variance (ANOVA) was performed for each parameter of the AIF. Additionally, the average amplitude, AUC, BAT, and TTP with 95% confidence intervals were compared between centers, where non-overlapping confidence intervals imply a statistically significant difference. Significant differences between groups of scanner manufacturers were also tested for with a one-way ANOVA for each parameter of the AIF. In these statistical calculations, AIFs with outliers in any of its parameters were excluded, where an outlier was defined as a data point more than 1.5 times the interquartile range below the first quartile or above the third quartile.

To indicate the variation of the AIF further, the proportion of explained variance, i.e., the sum of squares between groups divided by the sum of squares total, was determined for each parameter of the AIF. These proportions indicate how much of the variation is due to the center of admission and were compared to see which parameters would benefit the most from harmonization. Four centers were chosen as representative of the variation in AIF that results from the admission center. Although we were unable to retrieve all injection protocols retrospectively, these four centers were able to provide us with the injection protocols at the time of the DUST study.

### Design of simulation study

To assess the clinical impact of the variation in AIF between centers, a simulation study was performed demonstrating the effect on the estimated infarct core and penumbra volumes. The average AIF curve of each chosen center served as input for an updated version of an anthropomorphic digital phantom [11]. These center-specific average AIFs were constructed by aligning the peak of each individual patient curve before averaging these curves. If necessary, padding was performed by repeating the endpoints of the aligned AIFs.

We simulated a standard scanning protocol for the phantoms, consisting of 25 acquisitions at 80 kVp and 150 mAs for a total duration of 48 s with a 2-s interval between acquisitions and a slice thickness of 5 mm. In the first series of phantoms, a small infarct core with penumbra (8-mL core and 48-mL penumbra) was included in the right hemisphere, and in a second series a large infarct core with penumbra (26-mL core and 243-mL penumbra) was included in the right hemisphere. In both series, ten identical phantoms were generated for each of the four AIFs but with different randomly generated noise realizations, so we could take into account the influence of noise on the parameters [11].

### CTP analysis of digital phantoms

Because the AIF might affect infarct quantification differently depending on the perfusion software, the images were analyzed both with a commercial method (“Arrival Time Sensitive”) in IntelliSpace Portal (ISP; Brain Perfusion, IntelliSpace Portal 10.1, Philips Healthcare) and with in-house developed software based on a bSVD method, currently considered the clinical state-of-the-art to perform deconvolution on CTP data [4].

In ISP, the phantoms were automatically processed (filtering and automatic AIF/VOF selection) using proprietary methods. Factory default thresholds were applied to estimate volumes of the infarct core and penumbra, where infarct core is defined as tissue with a relative mean transit time (rMTT) > 150% and cerebral blood volume (CBV) < 2.0 mL/100g, and penumbra as tissue with rMTT > 150% and CBV > 2.0 mL/100g. For each set of ten noise realizations, the estimated core and penumbra volumes were displayed in a boxplot.

The in-house deconvolution software automatically determined the AIF of each phantom from the CTP image before filtering, using the method that was described earlier. After this, a bilateral filter with an isotropic spatial kernel of 3 mm and an intensity kernel of 20 HU was applied to filter the phantom noise realizations. Perfusion analysis was performed following a bSVD deconvolution method [12]. The infarct core was defined as tissue with a relative cerebral blood flow (rCBF) < 20%, and penumbra as tissue with a rCBF between 20 and 45%. These thresholds were found by maximizing the Dice similarity coefficient between the predicted and known regions for the core and penumbra of the digital phantom. The predicted core and penumbra volumes for each center were displayed in a boxplot for each set of the noise realizations.

### Statistical analysis of volumes

The mean estimated core and penumbra volumes for each center were compared using ANOVA for both of the processing methods. The mean mismatch, defined as the penumbra volume divided by the sum of the penumbra volume and core volume, was compared in the same way. In case of statistical significance, the volumes and mismatch were tested post hoc with Tukey’s honest significant difference test. The level of significance was defined as a two-tailed *p* < 0.05.

## Results

All DUST participants (*n* = 1422) gave informed consent for the use of their clinical and imaging data. Eleven of the acquired scans were excluded due to problems in registration of the data. The scanning protocols, reconstructed from the DICOM metadata of the remaining scans, can be found in the Supplementary Material. Dismissing scans that deviated from the general protocol of their respective centers resulted in another 17 exclusions so that a total of 1394 scans were analyzed. When analyzing the scanning protocols, we found that center F changed their scanning protocol at some point during the study, resulting in two distinct scanning protocols. Therefore, we split up this center into centers F1 and F2, corresponding to a scan acquisition with 1-s and 2-s intervals, respectively.

### Analysis of patient AIFs

**Fig. 3:**
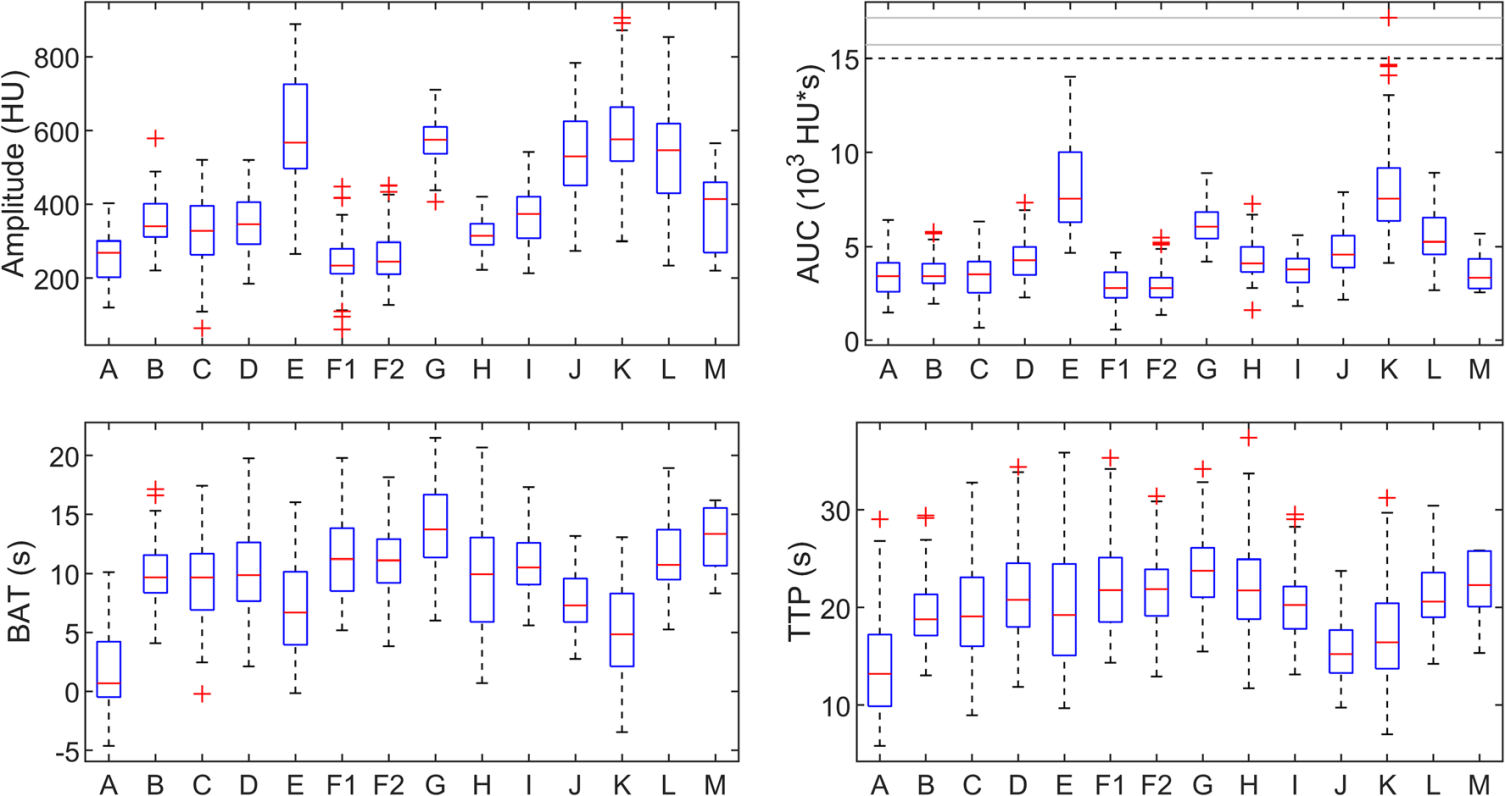
Boxplots of the parameters characterizing the arterial input function per stroke center. Outliers are depicted as red crosses. Extreme values were clipped, retaining the relative order, to avoid compressed boxplots

The boxplots of the amplitude, AUC, BAT, and TTP are shown in Fig. 3. These show a variation within centers, which can be explained by patient variability [6, 7], as well as between centers. Overall, the amplitude yields approximate interquartile ranges of 200–750 HU, the AUC of 2500–10,000 HU·s, the BAT of 0–17 s, and the TTP of 10–26 s.

The amplitude, AUC, BAT, and TTP each differed significantly between centers (all with *p* < 0.001). An indication of which centers differed significantly can be found in the Supplementary Material. For three groups of scanner manufacturers (i.e., Canon, Philips, and Siemens), no significant differences were found for the amplitude (*p* = 0.36), AUC (*p* = 0.20), BAT (*p* = 0.17), or TTP (*p* = 0.25). A specification of which centers were grouped, according to their scanner manufacturer, can be found in the Supplementary Material.

**Fig. 4:**
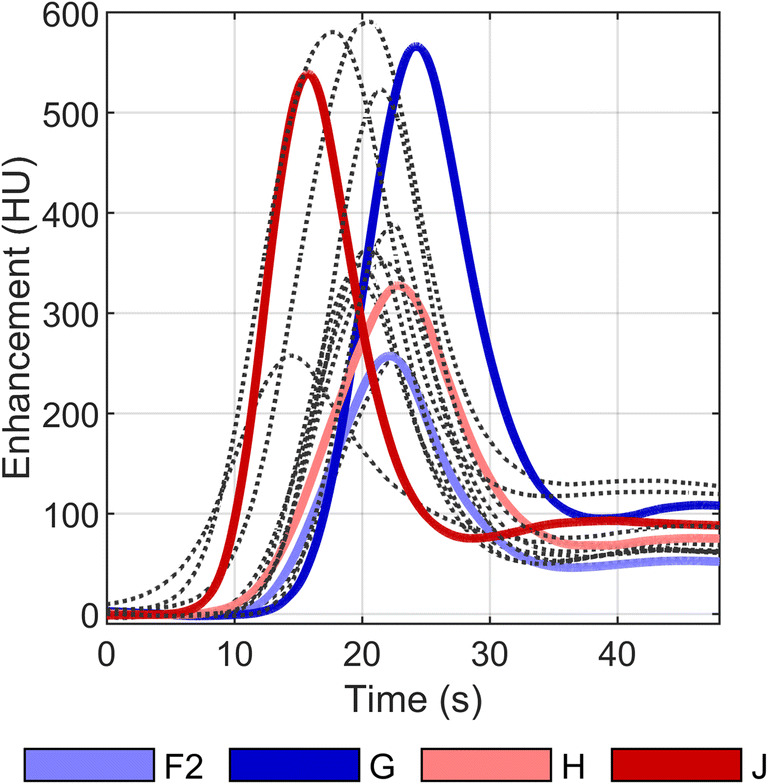
The average arterial input function (AIF) per stroke center. The four colored curves served as input for an anthropomorphic digital phantom. The dotted black curves are the AIFs of the other centers. Red curves have comparable AUCs and blue curves comparable widths. Light curves have a low amplitude and dark curves a high amplitude

The proportion of explained variance for the amplitude was 64.6%, for the AUC 63.3%, for the BAT 37.1%, and for the TTP 22.4%. Based on the amplitude and AUC, the centers F2 (light blue in the figures), G (dark blue in the figures), H (light red in the figures), and J (dark red in the figures) were selected for the simulation study, as they represent a large range in AIF. Their average AIF curves can be found in Fig. 4. The dark-colored curves have a high amplitude and the light-colored curves a low amplitude. Blue curves have a comparable width and red curves have a comparable AUC. The injection protocols of these centers are given in Table 1.

**Table 1:** Summary of the average parameters characterizing the arterial input functions selected to generate the anthropomorphic digital phantoms

Center	Solution for injection	Concentration (mg I/mL)	Volume (mL)	Injection rate (mL/s)
F2	Ultravist	300	40	6
G	Xenetix	300	60	5.5
H	Iomeron	300	35	6
J	Iomeron	400	40	6

### CTP analysis of digital phantoms

**Fig. 5:**
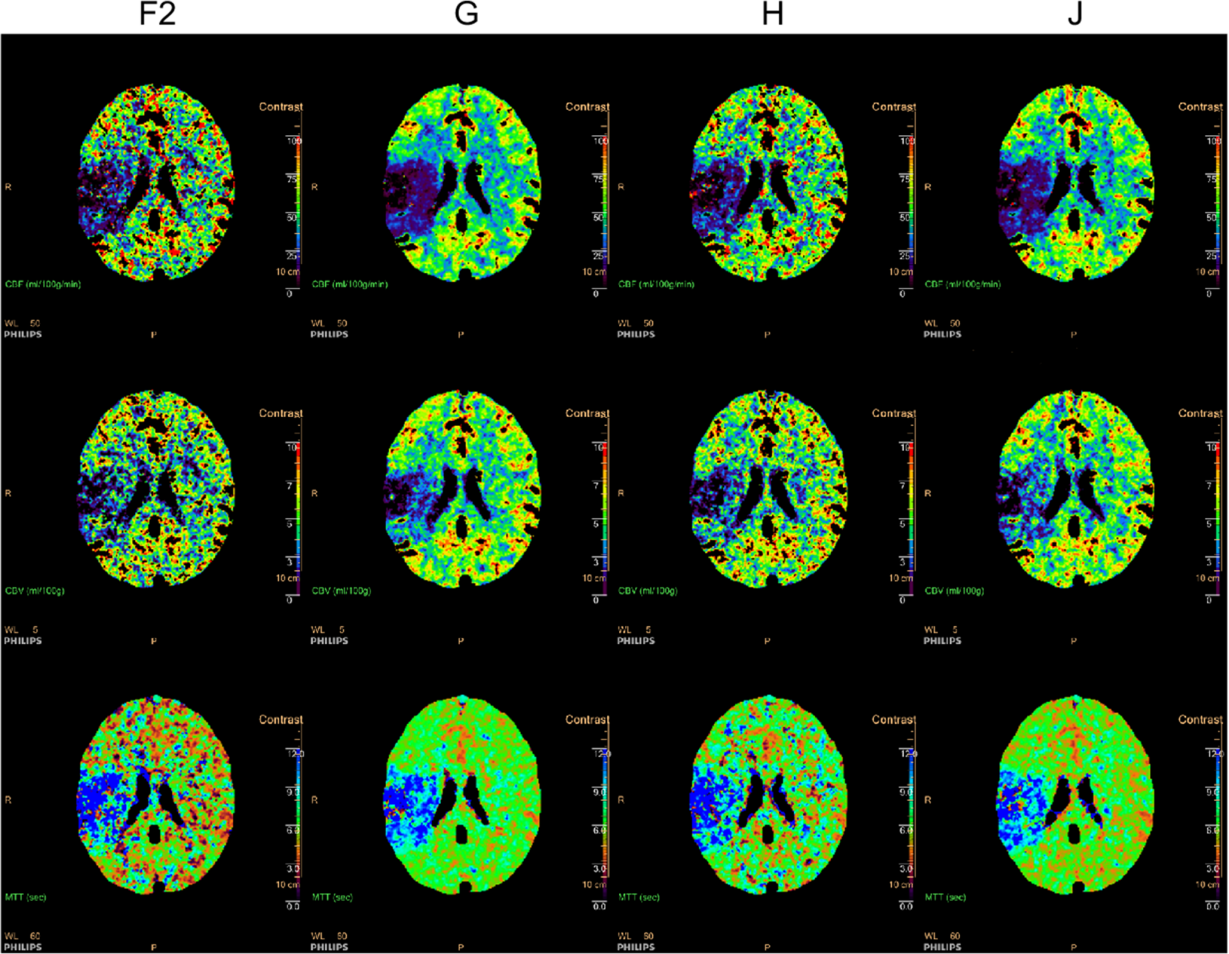
Parameter maps of one of the noise realizations of the anthropomorphic digital phantom with a small infarct for each of the four selected arterial input functions obtained from ISP. The cerebral blood flow (CBF; upper row) is in mL/100g/min, the cerebral blood volume (CBV; middle row) in mL/100g, and the mean transit time (MTT; bottom row) in seconds

**Fig. 6:**
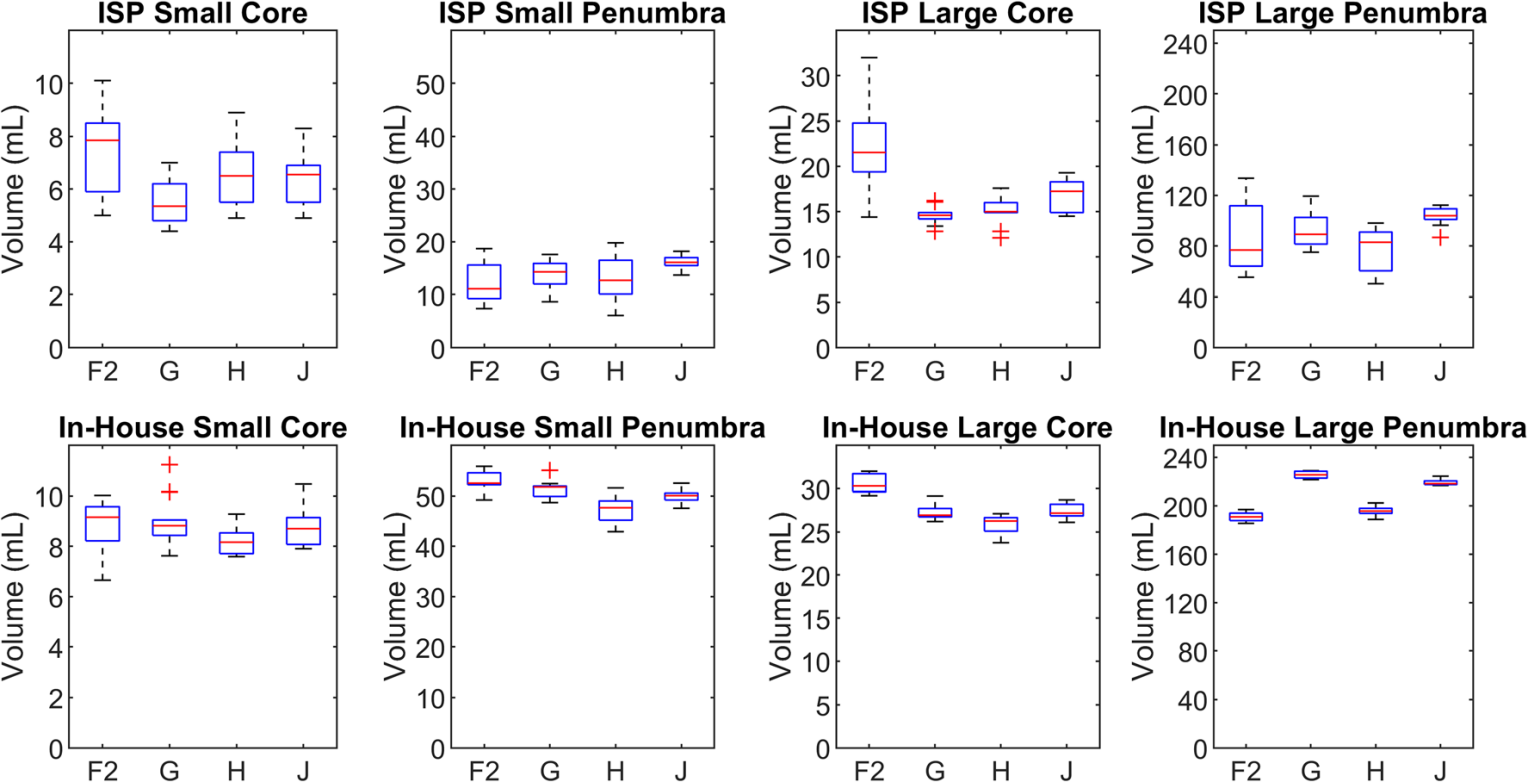
Boxplots of the estimated core and penumbra volumes for a small infarct (8***-***mL core and 48***-***mL penumbra) and a large infarct (26***-***mL core and 243***-***mL penumbra) obtained from ISP (upper row) and the in-house software (bottom row) for the representative centers. Outliers are depicted as red crosses

As an example of the anthropomorphic digital phantom, the parameter maps of ISP for one of the ten noise realizations that include a small infarct are shown in Fig. 5. Boxplots of the estimated core and penumbra volumes of the small and large infarct for each of the selected centers are shown in Fig. 6. Their median and interquartile range are indicated, along with the mismatch, in Table 2. The median core volume differed between centers from 0.1 to 7.0 mL, the median penumbra volume differed between centers from 0.8 to 34.5 mL, and the median mismatch differed between centers from 0 to 8%.

**Table 2:** Median core volume, penumbra volume, and mismatch with the interquartile range in brackets. The mismatch is defined as the penumbra volume divided by the sum of the penumbra volume and the core volume

Infarct	Center	Core volume (mL)	Penumbra volume (mL)	Mismatch
Small (ISP)	F2	7.9 (5.9 – 8.5)	11.1 (9.3 – 15.6)	0.63 (0.57 – 0.65)
	G	5.4 (4.8 – 6.2)	14.3 (12.0 – 15.9)	0.70 (0.68 – 0.74)
	H	6.5 (5.5 – 7.4)	12.7 (10.1 – 16.5)	0.67 (0.59 – 0.69)
	J	6.6 (5.5 – 6.9)	16.1 (15.5 – 17.0)	0.71 (0.69 – 0.75)
Large (ISP)	F2	21.6 (19.4 – 24.8)	76.9 (64.1 – 111.8)	0.80 (0.78 – 0.82)
	G	14.6 (14.2 – 14.9)	89.3 (81.5 – 102.6)	0.86 (0.85 – 0.87)
	H	15.0 (14.9 – 16.0)	83.0 (60.4 – 91.0)	0.84 (0.80 – 0.85)
	J	17.3 (14.9 – 18.3)	104.0 (101.0 – 109.4)	0.86 (0.85 – 0.87)
Small (in-house)	F2	9.2 (8.2 – 9.6)	52.5 (52.1 – 54.5)	0.85 (0.84 – 0.86)
	G	8.8 (8.4 – 9.0)	51.7 (49.9 – 51.9)	0.85 (0.85 – 0.86)
	H	8.2 (7.7 – 8.5)	47.7 (45.2 – 49.0)	0.85 (0.85 – 0.86)
	J	8.7 (8.1 – 9.1)	50.1 (49.2 – 50.6)	0.85 (0.84 – 0.86)
Large (in-house)	F2	30.3 (29.6 – 31.7)	191.0 (187.9 – 194.1)	0.86 (0.86 – 0.87)
	G	26.9 (26.7 – 27.7)	225.5 (222.8 – 228.5)	0.89 (0.89 – 0.89)
	H	26.3 (25.1 – 26.6)	195.8 (193.9 – 198.1)	0.88 (0.88 – 0.89)
	J	27.2 (26.9 – 28.2)	218.2 (217.5 – 220.5)	0.89 (0.89 – 0.89)

### Statistical analysis of volumes

The analysis with ISP yielded significantly different mean infarct core volumes for the small infarct (*p* = 0.01) between center F2 and center G. It also yielded significantly different mean infarct core volumes for the large infarct (*p* < 0.001) between center F2 and the other three centers. Mean penumbra volumes were not significantly different between centers for the small infarct (*p* = 0.06). For the large infarct, mean penumbra volumes differed significantly (*p* = 0.03) between centers H and J. The mean mismatch was significantly different for the small infarct (*p* < 0.001) between center F2 and centers G and J as well as between center H and centers G and J. The mean mismatch was significantly different for the large infarct (*p* < 0.001) for each pair of centers except between centers G and J.

The analysis with the in-house processing method yielded no significantly different mean core volumes between centers for the small infarct (*p* = 0.22). A significant difference was found for the large infarct (*p* < 0.001) for each pair of centers except between centers G and J. Mean penumbra volumes were significantly different for the small infarct (*p* < 0.001) between center H and the other three centers as well as between center F2 and center J. Mean penumbra volumes were also significantly different for the large infarct (*p* < 0.001) for each pair of centers. The mean mismatch for the small infarct was not significantly different between the centers (*p* = 0.80). For the large infarct, the mean mismatch was significantly different (*p* < 0.001) between center F2 and the other three centers as well as between center G and center H.

## Discussion

This study explored the variation in contrast injection protocol between centers, as characterized by their average AIFs, in a large multicenter stroke study. Significant differences in the magnitude and timing of the AIF were found between centers. This variation is important as it influences the variability of CTP analyses in a multicenter study. Harmonization of the injection protocol, as correspondent with the proportions of explained variance, could reduce variation in the amplitude with 64.6%, in the AUC with 63.3%, in the BAT with 37.1%, and in the TTP with 22.4%. Significant differences in infarct quantification were found as a result of the variation in amplitude and AUC between the average AIFs.

In clinical practice, imaging-based treatment decisions result from a combination of non-enhanced CT, occlusion site (provided by CT angiography), and CTP. The variation in CTP contrast bolus is relevant for current clinical practice, where treatment decisions may be based on the infarct core volume and the mismatch for patients presenting beyond 6 h after symptom onset [2, 3] or for patients with wake-up stroke [13, 14]. Although our study considered the variation in CTP contrast bolus, CT angiography also requires a contrast bolus, which is likely to vary between centers.

The influence on the AIF of the injection protocol, e.g., contrast concentration, injection rate and injection volume, as well as some patient characteristics, e.g., patient weight and cardiac output, has already been studied using simulations [6]. For some of these parameters, the effect on the AIF has also been studied within patient groups [7] and, in addition, the effect on the perfusion parameters has been examined for the contrast concentration [8]. Our study reported the variation in AIF between centers in a harmonized multicenter study and showed the clinical impact this variation can have.

In part, this clinical impact depends on the perfusion software. Differences in volumetric prediction between the two methods may be ascribed to different filtering methods, algorithms, and definitions of infarct core and penumbra. Harmonization of these image processing steps to reduce the variability of CTP results in a multicenter study presents itself as a major and important challenge. Our study showed that, given the differences in volumetric predictions within each processing method, harmonization of the injection protocol is likewise important.

There were several limitations. One major limitation was that the average AIF had to be used as a surrogate for the injection protocol, while at the same time the AIF was affected by the patient’s physiology. We assumed that the AIF of each patient is determined by the combination of the patient’s physiology and the contrast injection, which is expected to be constant within each center. Moreover, we assumed that the average physiology of the patients admitted to each center was comparable between the centers. Therefore, the variation between the average AIFs was assumed to be the result of differences in contrast injection. This justifies the average AIF as a proxy for the injection protocol, even though the exact connection between the average AIF and parameters of the injection protocol remains unclear.

Second, we were unable to retrieve the injection protocols of all the centers at the time of patient inclusions. Under the assumption that the average physiology of the patients admitted to each center was comparable between centers, the average AIF served as a surrogate for the injection protocol to check adherence to the advised injection protocol. For the selected representative centers, the injection protocols were made available. These show that the injection protocol of the DUST study, which lacked a restriction on the contrast concentration, was not strictly adhered to with regard to bolus volume and injection rate.

Third, the perfusion maps used to study the clinical impact of the AIF were not generated from patient CTP data but from a digital phantom. This was done in order to keep the pre- and postprocessing constant, so we could isolate the influence of the AIF. Since centers perform their scans on different scanners with different protocols and software, the cause of discrepancies in patient perfusion data is more difficult to establish.

Fourth, our focus with respect to the clinical relevance of varying AIFs has been on the amplitude and AUC of the AIF. Harmonization of the BAT and TTP could also prove to be beneficial as it may allow for more easily implemented modulated scanning protocols [15] and minimization of the risk on truncation of tissue curves [16].

## Conclusion

In the present study, we have shown that the variation in CTP contrast injection protocol between centers results in significant differences in the magnitude and timing of the AIF. The variation in the magnitude of the AIF between centers was greater than that within centers. This variation results in significant differences in core and penumbra volume estimation, which should be acknowledged in present multicenter studies and is relevant for current clinical practice.

## Supporting Information

ESM 1 (DOCX 133 kb)

## Abbreviations

AIF: Arterial input function
ANOVA: Analysis of variance
ASPECTS: Alberta Stroke Program Early CT Score
AUC: Area under the curve
BAT: Bolus arrival time
bSVD: Block-circulant singular value decomposition
CBF: Cerebral blood flow
CBV: Cerebral blood volume
CTP: Computed tomography imaging
DUST: DUtch acute STroke (study)
ISP: IntelliSpace Portal
MTT: Mean transit time
rCBF: Relative cerebral blood flow
rMTT: Relative mean transit time
TTP: Time to peak
VOF: Venous output function

## Declarations

### Guarantor

The scientific guarantor of this publication is H.W.A.M. De Jong.

### Statistics and biometry

No complex statistical methods were necessary for this paper.

### Study subjects or cohorts overlap

Some study subjects or cohorts have been previously reported in:

van Seeters, T., Biessels, G. J., van der Schaaf, I. C., Dankbaar, J. W., Horsch, A. D., Luitse, M. J., … & Velthuis, B. K. (2014). Prediction of outcome in patients with suspected acute ischaemic stroke with CT perfusion and CT angiography: the Dutch acute stroke trial (DUST) study protocol. *BMC neurology*, *14*(1), 1-8.

Van Der Hoeven, E. J., Dankbaar, J. W., Algra, A., Vos, J. A., Niesten, J. M., Van Seeters, T., … & Velthuis, B. K. (2015). Additional diagnostic value of computed tomography perfusion for detection of acute ischemic stroke in the posterior circulation. *Stroke*, *46*(4), 1113-1115.

Hamming, A. M., van Walderveen, M. A., Mulder, I. A., van der Schaaf, I. C., Kappelle, L. J., Velthuis, B. K., … & Algra, A. (2019). Circle of Willis variations in migraine patients with ischemic stroke. *Brain and behavior*, *9*(3), e01223.

van Seeters, T., Biessels, G. J., Kappelle, L. J., Van Der Schaaf, I. C., Dankbaar, J. W., Horsch, A. D., … & Schonewille, W. J. (2015). The prognostic value of CT angiography and CT perfusion in acute ischemic stroke. *Cerebrovascular diseases*, *40*(5-6), 258-269.

van Seeters, T., Biessels, G. J., Kappelle, L. J., van der Schaaf, I. C., Dankbaar, J. W., Horsch, A. D., … & Schonewille, W. J. (2016). CT angiography and CT perfusion improve prediction of infarct volume in patients with anterior circulation stroke. *Neuroradiology*, *58*(4), 327-337.

Horsch, A. D., Bennink, E., van Seeters, T., Kappelle, L. J., van der Graaf, Y., Mali, W. P., … & DUST Investigators. (2018). Computed tomography perfusion derived blood-brain barrier permeability does not yet improve prediction of hemorrhagic transformation. *Cerebrovascular Diseases*, *45*(1-2), 26-32.

### Methodology

• Retrospective

• Observational

• Multicenter study
