# Supplementary material for: Variation in arterial input function in a large multicenter computed tomography perfusion study
Source: Eur Radiol. 2021 May 28;31(11):8317–25. doi: 10.1007/s00330-021-08067-6 (PMC8523411; doi:10.1007/s00330-021-08067-6)
Supplement: ESM 1 — (DOCX 133 kb) [file 330_2021_8067_MOESM1_ESM.docx]

Supplementary Material 1

Overview of the different scanning protocols (by approximation) of the stroke centers. Center J used spiral scanning. Centers E and K used a wide-detector scanner. For the centers with a wide-detector scanner, the protocols are (2 acquisitions (with 4 s interval), 12 acquisitions (2 s), and last 5 acquisitions (5 s)) for center E and (2 acquisitions (3 s), 12 acquisitions (2 s), 5 acquisitions (5 s)) for center K. The scanner manufacturer for each center has been indicated by ‘C’ for Canon, ‘P’ for Philips or ‘S’ for Siemens.

| Center | Slices | Acquisitions | kVp | mAs | Included | Excluded [based on] |
| --- | --- | --- | --- | --- | --- | --- |
| A [C] | 8 (4 mm) | 25 (2 s) | 120 | 70 | 35 | 3 [slices: 4 (8 mm)]  2 [kVp: 80; mAs: 150] |
| B [P] | 12 (5 mm) | 25 (2 s) | 80 | 150 | 76 | 0 |
| C [S] | 6 (4.8 mm)  12 (5 mm) | 25 (2 s)  25 (2 s) | 80  80 | 150  140 | 73  41 | 0 |
| D [P] | 12 (5 mm) | 25 (2 s) | 80 | 125 | 232 | 3 [slices: 8 (3.5 mm); acquisitions: 40 (1 s); kVp: 90; mAs: 150] |
| E [C] | 32 (5 mm) | 19 (variable, see caption) | 80 | 75/100/200 | 30 | 5 [slices: 6 (5 mm); acquisitions: 25 (2 s); mAs: 150] |
| F1 [P] | 8/12 (5 mm) | 50 (1 s) | 80 | 75 | 63 | 1 [acquisitions: 41 (1 s)] |
| F2 [P] | 8/12 (5 mm)  8/12 (5 mm) | 25 (2 s)  30 (2 s) | 80  80 | 150  150 | 226  32 | 0 |
| G [P] | 8 (5 mm) | 25 (2 s) | 80 | 200 | 74 | 0 |
| H [S] | 6 (4.8 mm) | 25 (2 s) | 80 | 150 | 54 | 0 |
| I [P] | 12 (5 mm) | 25 (2 s) | 80 | 120/125 | 124 | 0 |
| J [S] | 18 (4 mm)  15 (5 mm)  19 (4 mm)  25 (3 mm) | 32 (1.25 s)  34 (1.5 s)  34 (1.5 s)  34 (1.5 s) | 80  80  80  80 | 250  220  220  220 | 15  25  7  30 | 0 |
| K [C] | 32 (5 mm) | 19 (variable, see caption) | 80 | 100/120/225 | 189 | 0 |
| L [C] | 8 (5.4 mm) | 25 (2.5 s) | 80 | 150 | 62 | 1 [acquisitions: 33 (2 s)]  1 [acquisitions: 30 (2.1 s)]  1 [slices: 4 (5.4 mm)] |
| M [P] | 8 (5.3 mm) | 25 (2 s) | 80 | 150 | 6 | 0 |


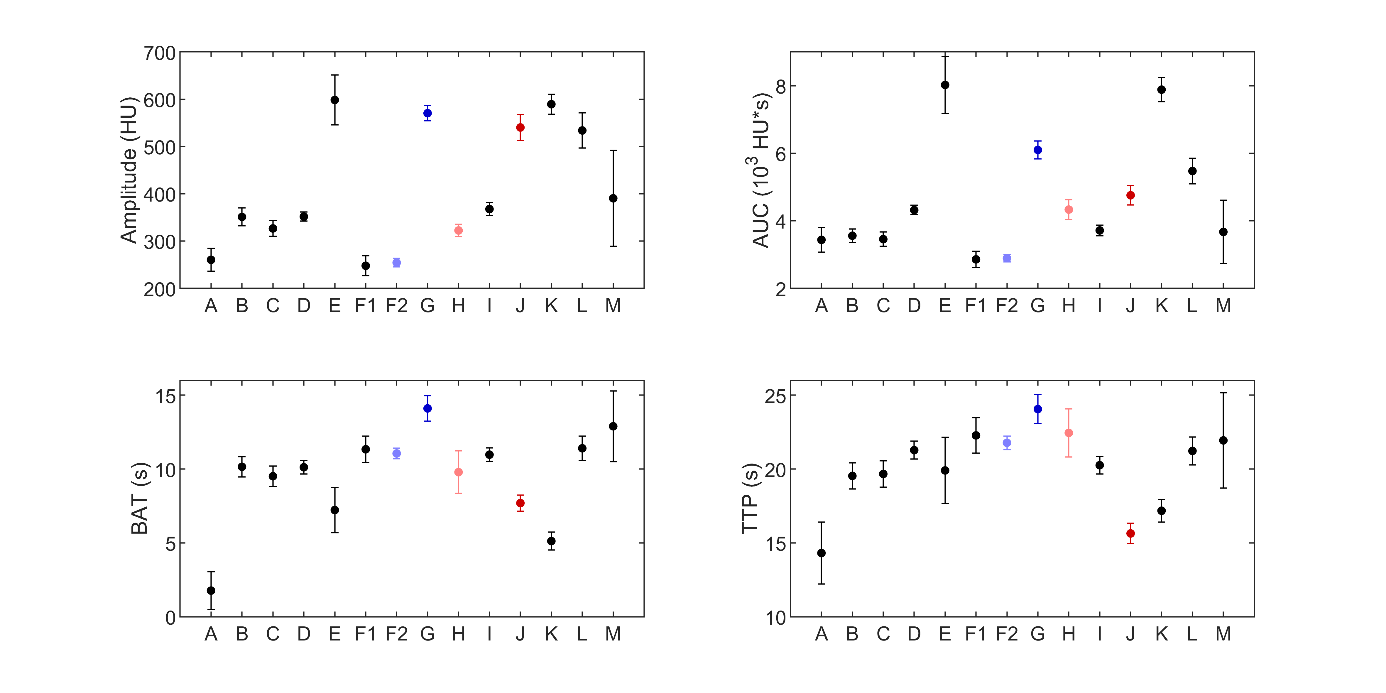


Supplementary Material 2

Average value and 95% confidence intervals of the parameters characterizing the AIF per stroke center. These show significant differences between stroke centers whenever confidence intervals do not overlap. The AIFs of the four colored centers were used as input for an anthropomorphic digital phantom.
